# First Report on Circulation of *Echinococcus ortleppi* in the one Humped Camel (*Camelus dromedaries*), Sudan

**DOI:** 10.1186/1746-6148-9-127

**Published:** 2013-06-25

**Authors:** Mohamed E Ahmed, Kamal H Eltom, Nasreen O Musa, Ibtisam A Ali, Fatima M Elamin, Martin P Grobusch, Imadeldin E Aradaib

**Affiliations:** 1 Molecular Biology Laboratory, Faculty of Veterinary Medicine, University of Khartoum, Khartoum, Republic of the Sudan; 2 Department of Surgery, Faculty of Medicine, Al-Neelain University, Khartoum, Republic of the Sudan; 3 Department of Internal Medicine, Faculty of Medicine, International University of Africa, Khartoum, Republic of the Sudan; 4 Unit of Animal Health and Safety of Animal Products, Institute for Studies and Promotion of Animal Exports, University of Khartoum, Khartoum, Republic of the Sudan; 5 Department of Infectious Diseases, Faculty of Medicine, Amsterdam Medical College, Amsterdam, The Netherlands

**Keywords:** *Echinococcus granulosus*, NADH 1 gene, cox1 gene, Genotypes, Phylogenetic analysis, Sudan

## Abstract

**Background:**

*Echinococcus granulosus* (EG) complex, the cause of cystic echinococcosis (CE), infects humans and several other animal species worldwide and hence the disease is of public health importance. Ten genetic variants, or genotypes designated as (G1-G10), are distributed worldwide based on genetic diversity. The objective of this study was to provide some sequence data and phylogeny of EG isolates recovered from the Sudanese one-humped camel *(Camelus dromedaries*). Fifty samples of hydatid cysts were collected from the one- humped camels *(Camelus dromedaries*) at Taboul slaughter house, central Sudan*.* DNAs were extracted from protoscolices and/or associated germinal layers of hydatid cysts using a commercial kit. The mitochondrial NADH dehydrogenase subunit 1 (NADH1) gene and the cytochrome C oxidase subunit 1 (cox1) gene were used as targets for polymerase chain reaction (PCR) amplification. The PCR products were purified and partial sequences were generated. Sequences were further examined by sequence analysis and subsequent phylogeny to compare these sequences to those from known strains of EG circulating globally.

**Results:**

The identity of the PCR products were confirmed as NADH1 and cox1 nucleotide sequences using the Basic Local Alignment Search Tool (BLAST) of NCBI (National Center for Biotechnology Information, Bethesda, MD). The phylogenetic analysis showed that 98% (n = 49) of the isolates clustered with *Echinococcus canadensis* genotype 6 (G6), whereas only one isolate (2%) clustered with *Echinococcus ortleppi* (G5).

**Conclusions:**

This investigation expands on the existing sequence data generated from EG isolates recovered from camel in the Sudan. The circulation of the cattle genotype (G5) in the one-humped camel is reported here for the first time.

## Background

The larval stage of *Echinococcus granulosus* complex (EG) causes cystic echinococcosis (CE) in domestic livestock and humans. Because of the involvement of the vital organs, CE in humans is considered a critical public health problem. In addition to human health concerns, infections in cattle breeding areas may result in economic losses [1]. Moreover, CE represents one of the neglected tropical diseases, especially in the Sub- Saharan Africa [2]. In the Sudan, many reports of cystic echinococcosis have been described in humans and animals [3-12]. In addition to its importance as a major public health problem in the country, CE is also considered as one of the major causes of condemnation of sheep carcasses during meat inspection [13]. Currently, 10 distinct genotypes of EG designated as G1-G10 have been described worldwide on the basis of genetic diversity related to nucleotide sequences of the mitochondrial cytochrome C oxidase subunit 1 (cox1) and NADH dehydrogenase subunit 1 (NADH 1) genes. These different genotypes are associated with distinct intermediate hosts including sheep, pigs, cattle, horses, camels, goats and cervids [14-25]. Of the ten genotypes of EG, the sheep (G1), the cattle (G5) and the camel (G6) strains were reported in humans and livestock in the Sudan [8-10,26-28]. Recent epidemiological studies indicated that the camel strain (G6) was the most prevalent strain in Sudan [10,27,28]. EG complex comprises a number of intra-specific variants, strains or genotypes at the genetic level [29-32]. It was suggested that the extensive intra-specific genetic variation of *E. granulosus* could be better understood within the context of variations in the life cycle pattern [29]. In addition, different genotypes would probably exhibit different antigenicity, transmission profiles, and sensitivity to chemotherapeutic agents as well as different pathological consequences [29,33,34]. These biological variations should be considered in developing vaccines, diagnostic kits and pharmacological therapies for control of CE. Therefore, due to epidemiological implementation and control strategies, it is essential that circulating EG genotypes in a given area of endemicity should be clearly defined [30,35]. In this study, hydatid cysts recovered from the one- humped camel (*Camelus dromedaries*) in an endemic area of Tamboul, Central Sudan, were defined by genetic studies and subsequent phylogenetic analysis. The molecular characterization was made possible by targeting fragments of the mitochondrial NADH 1 and cox1 subunit 1 genes to define the circulating genetic variants in the hyper endemic area of Tmboul, Central Sudan.

## Methods

### Collection of samples

Hydatid cysts (n = 50) were collected over a period of one month from camel at the slaughterhouse of Tamboul, a village located at the camel producing region of Central Sudan. This slaughterhouse represents one of the major abattoirs of camel in the Sudan. The hydatid cysts were obtained from camels soon after slaughtering and transferred in thermo- flasks to the Molecular Biology Laboratory at the Faculty of Veterinary Medicine, University of Khartoum, for processing and molecular characterization studies. The cysts were mainly located in the lung (n = 45) and the rest were found in the liver (n = 5). All cysts were fertile and measured 2–10 cm in diameter. Hydatid cysts containing protoscolices and associated germinal layers were aspirated with sterile needles. The aspirates were transferred to clean sterile 50 ml tubes to which 70% alcohol was added as preservative.

### DNA Extraction from intact cysts

The suspensions containing protoscolices and/or associated germinal layers were washed in nucleic acid free water to remove excess alcohol. Extraction of DNA from hydatid cysts was made possible using a commercially available QIAamp tissue kit (QIAGEN, Hilden, Germany) according to the manufacturer’s instructions. Briefly, 200 μl of the suspended aspirate, 20 μl of proteinase K stock solution, and 200 μl of lysing buffer were pipetted into 1.5 ml eppendorf tube. The mixture was incubated at 37°C for 1 h and then at 70°C for 30 min before the addition of 200 μl of absolute alcohol and mixing by vortexing. The mixture was then transferred to the QIAamp spin column placed in a clean 2 ml collection tube and centrifuged at 8000 RPM in MiniSpin centrifuge (Eppendorf, Wesseling-Berzdorf, Germany) for 1 min at room temperature. The QIAamp spin column was washed twice with 500 μl of the washing buffers by spinning for 1 min. The QIAamp spin column was placed in a clean 1.5 ml eppendorf tube and the DNA was eluted with 200 μl of double distilled water preheated at 70°C. Maximum DNA yield was obtained by spinning at 12,000 RPM for 1 min at room temperature. From the suspended nucleic acid 5 μl was used in the PCR amplification.

### Selection of primers

The primers were selected from mitochondrial NADH dehydrogenase subunit 1 (NADH 1) gene and cytochrome C oxidase subunit 1 gene (cox1) gene. The NADH 1 primers used in this study were basically described previously [17]. For the first amplification step, a pair of outer primers JBl1: 5' AGATTCGTAAGGGGCCTAATA 3' and JB12: 5' ACCACTAACTAATTCACTTTC 3' were used to amplify a 530 bp PCR product from EG isolates. A pair of internal sequencing primers JB11.5: 5' TTATGGTAGATATTATAG 3' and JB12.5: 5' CACACACATAAAACAAGC 3' designed to conserved segments of the EG NADH 1 sequences, were used to generate a 471 bp PCR product. The cox1 primers used in this study include a forward primer CO1: 5' GAG GTT TAT TTT TTT GGG CAT CCT 3' and a reverse primer CO1: 5' TAA AGA AAG ATA ATG AAA ATG 3' as described before [17]. CO1 and CO2 primers would produce a 415 bp specific PCR product.

### Polymerase chain reaction

A stock buffered solution containing 150 μl 10x PCR buffer, 100 μl of 25 mM MgCl_2_, 12.5 μl of each dATP, dTTP, dGTP and dCTP at a concentration of 10 mM was prepared in 1.5 ml eppendorf tube. The primers were used at a concentration of 20 pg/μl, and double distilled water was added to bring the volume of the stock buffer solution to 1.5 ml. Each 0.5 ml PCR reaction tube contained 2 μl of the primers, 1 μl (5.0 U) of Taq DNA polymerase (QIAGEN), 5.0 μl of the target DNA and 42 μl of the stock buffered solution. For nested PCR, 2 μl of the primary PCR product was used as DNA template. The thermal cycling profiles were as follows: a 2 min initial incubation at 95°C, followed by 40 cycles of 95°C for 1 min, 55°C for 30 sec and 72°C for 45 sec, and a final incubation at 72°C for 10 min. Thermal profiles were performed on a Techne TC-412 thermal cycler (Techne, Staffordshire, UK). Following amplification, 15 μl from each PCR containing amplified products were loaded onto gels of 1.0% agarose and electrophoresed for 1 h. The gels were stained with ethidium bromide and the PCR products were easily identified following visualization under UV light.

### Sequence analysis and construction of phylogenetic tree

The primary PCR products were purified using QIAquick PCR purification kit (QIAGEN) and sent for sequencing to commercial companies (Seqlab, Göttingen, Germany, and Macrogen, Seoul, Korea). Resulted sequences were edited and aligned using BioEdit software (Ibis Biosciences, Carlsbad, CA, USA). The Basic Local Alignment Search Tool (BLAST) of NCBI (National Center for Biotechnology Information, Bethesda, MD, USA) was used to confirm the identity of the generated sequences in relation to the GenBank nucleotide database. The sequences were then aligned with the corresponding regions of NADH 1 and cox1 subunit genes of known genotypes from other countries. The phylogenetic trees were constructed using Unweighted Pair Group Method with Arithmetic mean (UPGMA) implemented in MEGA software version 5.0 [36]. The country of origin, the GenBank accession numbers and the genotype were given for each EG isolate when available. Bootstrap analysis of 1000 replicates was applied and values were given at relevant nodes of the constructed tree. Corresponding nucleotide sequences of NADH 1 and cox 1 of *Taenia multiceps* with GenBank accession numbers HM143887.1 and JX535576.1, respectively; were used as out groups in the constructed phylogenetic trees.

## Results

### Polymerase chain reaction (PCR)

The PCR-based assay with primers specific for NADH1 and cox1 genes yielded amplification products from of all of the fifty hydatid cysts obtained from naturally infected camels. For NADH1, the outer pair of primers produced a primary 530 bp PCR product and the nested primers produced a 471 bp PCR product. Cox 1 primers produced 415 bp PCR products.

### Sequence analysis and phylogenetic relationship

**Figure 1:**
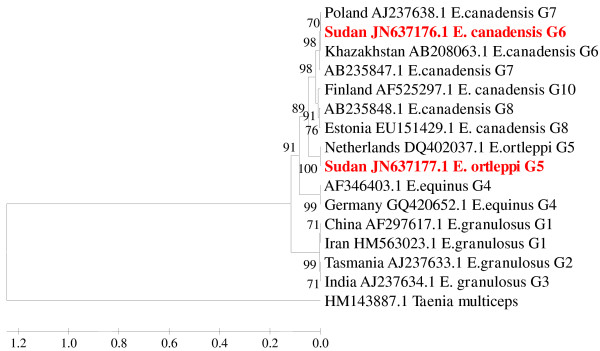
**Phylogenetic relationship of *Echinococcus granulosus*-complex genotypes recovered from Sudanese camels and other genotypes identified globally.** Partial NADH dehydrogenase subunit 1 gene sequences generated from EG isolates were aligned with sequences of other EG isolates from different parts of the world. Sequences were analyzed with the BioEdit software (Ibis Biosciences, Carlsbad, CA, USA). The phylogenetic tree was constructed using Unweighted Pair Group Method with Arithmetic mean (UPGMA) implemented in MEGA software version 5 [36]. Bootstrap values were calculated from analysis of 1000 replicates of the data set, and values greater than 50% are indicated at the appropriate nodes. Each EG isolate is designated by the country of origin, GenBank accession number and the genotype of the isolate when available. The GenBank accession numbers (JN637176, JN637177) were given for the Sudan isolates of genotype 5 (*E. ortleppi*) and genotype 6 (*E. canadensis*), respectively. Corresponding nucleotide sequence of NADH 1 of *Taenia multiceps*, GenBank accession number HM143887.1, is used as an out group in the constructed phylogenetic tree. The strains from Sudan are highlighted in red color for clarity.

**Figure 2:**
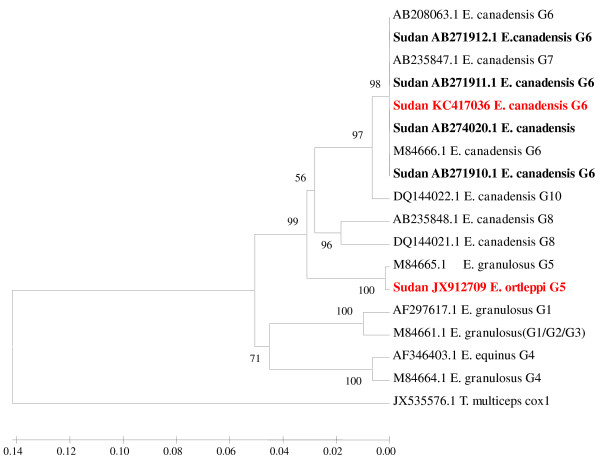
**Phylogenetic relationship of *Echinococcus granulosus-*complex genotypes recovered from Sudanese camels and other genotypes identified globally.** Partial Cox 1 gene sequences generated from EG isolates, used in this study, were aligned with sequences of other EG isolates from different parts of the world. Sequences were analyzed with BioEdit software (Ibis Biosciences, Carlsbad, CA, USA). The phylogenetic tree was constructed using Unweighted Pair Group Method with Arithmetic mean (UPGMA) implemented in MEGA software version 5 [36]. Bootstrap values were calculated from analysis of 1000 replicates of the data set, and values greater than 50% are indicated at the appropriate nodes. Each EG isolate is designated by the country of origin, GenBank accession number and the genotype of the isolate when available. The GenBank accession numbers (KC417036, JX912709) were given for *E. ortleppi* (G5) and *E. canadensis* (G6), respectively. Corresponding nucleotide sequence of cox 1 of *Taenia multiceps*, GenBank accession number JX535576.1, is used as an out group in the constructed phylogenetic trees. The strains from Sudan are bolded and those described in this study are highlighted in red color for clarity.

The sequences obtained from the PCR products were found to align with corresponding regions for NADH1 and cox1 genes in the GenBank confirming the cysts to contain the EG complex. Aligned with BioEdit, partial sequences for NADH 1 and cox1 showed 100% homology among 49 out of 50 EG isolates recovered in this study. To investigate for the relationship between these EG isolates and the other EG genotypes identified globally, phylogenetic trees were constructed (Figures 1 and 2). Forty nine (98%) of EG isolates (represented by one sequence in the tree) clustered with *E. canadensis* genotype 6 (G6), the camel genotype, of EG complex obtained from other parts of the world with a strong bootstrap (1000 replicates). However, only one EG isolate (2%) clustered with *E. ortleppi*, the cattle genotype (G5). The partial sequences of NADH 1 gene representing the camel genotype (G6) and of the cattle genotype (G5) were submitted to the GenBank under accession numbers JN637176, JN637177, respectively. The partial sequences generated from mitochondrial cytochrome C oxidase subunit 1 (cox1) for G6 and G5 strains were submitted to the GenBank under accession numbers KC417036, JX912709, respectively.

## Discussion

In Sudan, camels are owned by migratory pastoralists as a source of milk, meat, riding animals, as well as sign of wealth. Extensive research has been conducted to evaluate the role played by camels in transmission of parasitic infections with special emphasis on cystic echinococosis [9,10,37]. The camel strain (G6) was reported to be the most prevalent genotype of EG in the Sudan, and that camels seem to play an important role in the transmission cycle of the parasite and the epidemiology of the disease. In contrast, the majority of the Sudanese ecotypes of desert sheep and Nubian goats seem to harbor calcified or infertile cysts of EG-complex [9,10,38]. It was, therefore, suggested that sheep and goats have natural resistance to infection with EG-complex. However, this assumption requires further investigation. To advance beyond the current knowledge of the epidemiology of the disease in camel, attempts were made to better understand the life cycle and molecular epidemiology of EG isolates circulating in different parts of the Sudan. An initial step in controlling the life cycle of EG and minimizing infections is to determine the genotype(s) involved in the transmission cycle. In a previous study, different genotypes including G5 and G6 were confirmed in the Sudan by partial sequence analysis of the mitochondrial cytochrome oxidase 1 (cox1) and NADH dehydrogenase subunit 1 genes [8]. However, no phylogenetic study was conducted to determine the phylogenetic relationship of the identified genotypes of EG in the Sudan. In this study, the phylogenetic analysis illustrated that *Echinococcus canadensis* genotype 6 (G6) is the most infectious and widespread genotype in the Sudan, confirming the results of our previous studies [8,27]. Nevertheless, the present study indicated that *Echinococcus ortleppi,* the cattle genotype 5 (G5) should equally be considered as an infectious form of EG-complex in the one humped camels in Sudan. Sequence analysis and subsequent phylogenetic studies of fragments of the mitochondrial NADH subunit 1 gene indicated the presence of a single isolate of the cattle genotype (G5). However, the remaining 49 (98%) isolates belonged to *E. canadensis* (G6). Similar results were obtained when constructing phylogenetic trees using partial sequences derived from cox1 subunit gene, thus confirming the identity of the presence of cattle genotype (G5). This finding provides an alarming evidence for the circulation of the cattle genotype (G5) in the one-humped camels. It is, however, uncertain whether the G5-infected camel was maintained in Tamboul area, Central Sudan, or brought from a neighboring area for the purpose of slaughtering. The circulation of a major variant (G6) in Sudanese camels suggests that specific mechanisms are responsible for its persistence in the endemic area of Tamboul, Central Sudan. This is probably due to close relationships between dogs and camels in the study area [39]. In rural communities with resource-poor settings, such as this study area, the practice of animal slaughtering is usually performed in the open space. Under these conditions, dogs would have free access to feed on livestock viscera, which may harbor hydatid cysts, the infective stage. Therefore, it is believed that this practice of livestock slaughtering could effectively contribute towards the persistence of the camel genotype (G6) in the study area.

The addition of EG strains sequences from Sudan enhances our understanding of the expansion and, to some degree, maintenance of the parasites in the intermediate hosts. Ongoing surveillance and EG strains characterization should also aid in determining the distribution of this cestode parasite in the country. As more sequencing data and prediction tools become more accurate and available, these data will provide the public health authorities an opportunity to anticipate and prepare for treatment and subsequent control programs for the disease.

## Conclusion

In conclusion, the result of this study indicates the circulation of *E. ortleppi*, the cattle genotype (G5) in the one humped camel in the Sudan for the first time. Therefore, the G5 strain should be considered during epidemiological surveys for this important parasitic infection in Sudan. In addition, this investigation expands on the existing data on sequences generated from EG isolates recovered from the one humped camel in the endemic area of Tamboul, Central Sudan.

## Competing interests

The authors declare that they have no competing interests.

## Authors’ contributions

MEA collected hydatid cyst samples, extracted the DNA and optimized the polymerase chain reaction-based detection assay; KAE analyzed the sequences and designed the study; IAA and FME designed the experiment; NOM, edited the sequences and helped with experimental design. IEA designed the experiment and prepared the final manuscript. All authors read and approved the final version of the manuscript.
